# Abnormal Gray Matter Structural Networks in Idiopathic Normal Pressure Hydrocephalus

**DOI:** 10.3389/fnagi.2018.00356

**Published:** 2018-11-15

**Authors:** Le-Kang Yin, Jia-Jun Zheng, Jia-Qi Tian, Xiao-Zhu Hao, Chan-Chan Li, Jian-Ding Ye, Yu-Xuan Zhang, Hong Yu, Yan-Mei Yang

**Affiliations:** ^1^Department of Radiology, Huashan Hospital of Fudan University, Shanghai, China; ^2^Department of Radiology, Shanghai Chest Hospital, Shanghai Jiao Tong University, Shanghai, China; ^3^Department of Neurosurgery, Huashan Hospital of Fudan University, Shanghai, China; ^4^Medical Biology Centre, School of Pharmacy, Faculty of Medicine, Health and Life Sciences, Queen’s University of Belfast, Belfast, United Kingdom; ^5^Department of Radiology, Shanghai East Hospital, Tongji University School of Medicine, Shanghai, China

**Keywords:** normal pressure hydrocephalus, structural networks, gray matter, network modularity, magnetic resonance imaging

## Abstract

**Purpose**: Idiopathic normal pressure hydrocephalus (iNPH) is known as a treatable form of dementia. Network analysis is emerging as a useful method to study neurological disorder diseases. No study has examined changes of structural brain networks of iNPH patients. We aimed to investigate alterations in the gray matter (GM) structural network of iNPH patients compared with normal elderly volunteers.

**Materials and Methods**: Structural networks were reconstructed using covariance between regional GM volumes extracted from three-dimensional T1-weighted images of 29 possible iNPH patients and 30 demographically similar normal-control (NC) participants and compared with each other.

**Results**: Global network modularity was significantly larger in the iNPH network (*P* < 0.05). Global network measures were not significantly different between the two networks (*P* > 0.05). Regional network analysis demonstrated eight nodes with significantly decreased betweenness located in the bilateral frontal, right temporal, right insula and right posterior cingulate regions, whereas only the left anterior cingulate was detected with significantly larger betweenness. The hubs of the iNPH network were mostly located in temporal areas and the limbic lobe, those of the NC network were mainly located in frontal areas.

**Conclusions**: Network analysis was a promising method to study iNPH. Increased network modularity of the iNPH group was detected here, and modularity analysis should be paid much attention to explore the biomarker to select shunting-responsive patients.

## Introduction

Idiopathic normal pressure hydrocephalus (iNPH) is a chronic communicating hydrocephalus with a characteristic triad of gait disturbance, dementia and incontinence. First described by Hakim and Adams in 1965 (Adams et al., 1965), it is known as a treatable form of dementia as symptoms could relieve somewhat after extrathecal cerebrospinal fluid (CSF) shunting. The diagnosis of iNPH is made on the basis of clinical symptoms, brain imaging revealing ventricular enlargement and data from other invasive methods such as lumbar puncture and external lumbar drainage according to the International iNPH Guidelines (Relkin et al., 2005). A number of neuroimaging methods have been used to explore the structural and functional changes of iNPH patients, and to search for predictive biomarkers for the outcome of shunting (Børgesen and Gjerris, 1982; Tarnaris et al., 2009; Lenfeldt et al., 2011). However, little is known about the pathogenesis and there is still lack of effective methods to select potential responsive patients.

In recent years, the graph theory was used to examine brain structural or functional network organization. The human brain is a complex and interacting network with nontrivial topological properties (Sporns et al., 2004; Eguíluz et al., 2005; Stam et al., 2007). The characterization of the underlying architectures of such a network is an important issue in neuroscience (He et al., 2007). Many chronic brain disorders with cognitive, emotional, perceptual, and motor symptoms are associated with abnormalities in the brain network organization (Bullmore and Sporns, 2012). Previous studies have demonstrated changes in neuronal connectivity between different brain regions in several diseases associated with cognitive impairment, such as Alzheimer’s disease (Stam et al., 2007) and multiple sclerosis (He et al., 2009). This method provides a new insight into the mechanisms of neuropsychiatric disorders and helps us to find new imaging biomarkers to diagnose and monitor neurological diseases. Knowing that structural network provides the anatomical frame within which functional interactions took place and shapes the functional network. Indeed, alterations in default mode network connectivity of iNPH patients have been detected by using resting-state functional MRI (Khoo et al., 2016). Hence, a hypothesis was that abnormalities exist in the brain structural network of iNPH patients.

The present study aimed to use covariance between regional gray matter (GM) volumes on the basis of structural MRI data to construct a putative structural network of iNPH patients’ group. We then compare the structure network of the iNPH group with a normal-control (NC) group to investigate if there are any abnormalities in regional or global network measures.

## Materials and Methods

### Subjects

Between October 2013 and September 2016, 33 patients (age: 65.5 ± 9 years; range: 56–82 years) fulfilled the diagnosis of probable iNPH according to the International iNPH Guidelines (Relkin et al., 2005). They presented with dilated ventricles on brain images with an Evans’ Index being larger than 0.3, and had at least two of the triad of symptoms, namely gait disturbance, dementia, or urinary incontinence. The median intracranial pressure of the iNPH group was 110.0 mmH_2_O (range: 88–150 mmH_2_O; interquartile range: 21.5 mmH_2_O).

A total of 33 age- and gender-matched volunteers (the normal control or NC group; age: 62.6 ± 9.1 years; range: 54–81 years) were recruited and received MRI scanning that was the same as that performed for the iNPH group. All the control subjects were selected from a database that included healthy adults who attended screening in the hospital from January 2015 to June 2016. None of them had any evidence of focal brain lesions on routine MR images except for age-related brain atrophy and hyperintensities on T2-weighted images, and presented with a normal physical and neurological profile with no history of psychiatric disorders.

Fifteen iNPH patients and 20 controls were included in our earlier work that dealt with the changes of CSF flow though aqueduct (Yin et al., 2017). In contrast in this manuscript, we report the changes of the structural network. Eligible subjects were fully informed about the study and signed a consent form. This study was carried out in accordance with the recommendations of ethics committee of Huashan Hospital with written informed consent from all subjects. All subjects gave written informed consent in accordance with the Declaration of Helsinki. The protocol was approved by the ethics committee of Huashan Hospital of Fudan University.

### MRI Data Acquisition

All the subjects underwent MR scanning in a 3 Tesla MR unit (Magnetom Verio System; Siemens, Erlangen, Germany). The sagittal three-dimensional T1-weighted images were obtained using the magnetization-prepared rapid acquisition gradient-echo (MPRAGE) sequence, which covered the whole head. The sequence parameters were as follows: repetition time, 2,300 ms; echo time, 2.98 ms; flip angle, 9°; slice thickness, 1 mm; field of view, 256 × 256 mm; matrix, 256 × 256; pixel size, 1 × 1 mm.

### Preprocessing of MRI Data

Image preprocessing was performed using the VBM8 toolbox implemented within Statistical Parametric Mapping 8 (SPM8; Wellcome Department of Cognitive Neurology, London, United Kingdom). All MRI data were checked for artifacts and movement and aligned with the anterior commissure-posterior commissure (AC-PC) line manually. The images were segmented into GM, white matter (WM) and CSF segments. The GM images were nonlinearly normalized into standard Montreal Neurological Institute (MNI) space using an age- and gender-adjusted GM study-specific customized template created by the Template-O-Matic (TOM8) toolbox (Wilke et al., 2008). The images were then modulated to ensure that relative volumes of GM were preserved following the spatial normalization procedure. Sample homogeneity was checked to identify any outliers in the study population. Data of four participants in the iNPH group and three in the NC group were excluded from the analysis because of a covariance below 2 standard deviation (SD) and visual confirmation of a motion artifact. Total intracranial volume was calculated as the sum of GW, WM and CSF volumes obtained in the segmentation step.

### Network Node Definition

A total of 90 cortical and subcortical regions of interest (ROIs; Table 1), excluding the cerebellum, were generated from the Automated Anatomical Labeling (AAL) atlas using the Wake Forest University (WFU) PickAtlas Toolbox (Tzourio-Mazoyer et al., 2002). The ROIs were identical to those used in a previous graph analysis study by Fan et al. (2011). The ROIs were resliced to the same dimension as the tissue-segmented images obtained in the preprocessing step. Then the ROIs were used to mask the individual-modulated and normalized GM images and the average volume of each ROI was extracted using the REX toolbox^1^. A multivariable linear regression analysis was conducted at every ROI to control for the effects of age and total brain volume, with the residuals measuring the corrected GM volume for each subject and ROI (Bernhardt et al., 2011; Fan et al., 2011).

**Table 1:** List of anatomical descriptions of node used in the network.

Number of nodes	Anatomical description (Left/Right)
1/2	Precentral
3/4	Superior frontal gyrus, dorsolateral
5/6	Superior frontal gyrus, orbital part
7/8	Middle frontal
9/10	Middle frontal gyrus, orbital part
11/12	Inferior frontal, opercular part
13/14	Inferior frontal gyrus, triangular part
15/16	Inferior frontal gyrus, orbital part
17/18	Rolandic operculum
19/20	Supplementary motor area
21/22	Olfactory cortex
23/24	Superior frontal gyrus, medial
25/26	Superior frontal gyrus, medial orbital
27/28	Gyrus rectus
29/30	Insula
31/32	Anterior cingulum
33/34	Middle cingulum
35/36	Posterior cingulum
37/38	Hippocampus
39/40	Parahippocampal gyrus
41/42	Amygdala
43/44	Calcarine
45/46	Cuneus
47/48	Lingual gyrus
49/50	Superior occipital
51/52	Middle occipital
53/54	Inferior occipital
55/56	Fusiform
57/58	Postcentral
59/60	Superior parietal
61/62	Inferior parietal
63/64	Supramarginal
65/66	Angular
67/68	Precuneus
69/70	Paracentral lobule
71/72	Caudate
73/74	Lenticular nucleus, putamen
75/76	Lenticular nucleus, pallidum
77/78	Thalamus
79/80	Heschl gyrus
81/82	Superior temporal gyrus
83/84	Temporal pole: superior temporal gyrus
85/86	Middle temporal gyrus
87/88	Temporal pole: middle temporal gyrus
89/90	Inferior temporal gyrus

*Odd number represents the anatomical region located in the left cerebral hemisphere, and the even number represents that in the right hemisphere*.

### Construction of Structural Covariance Networks

**Figure 1:**
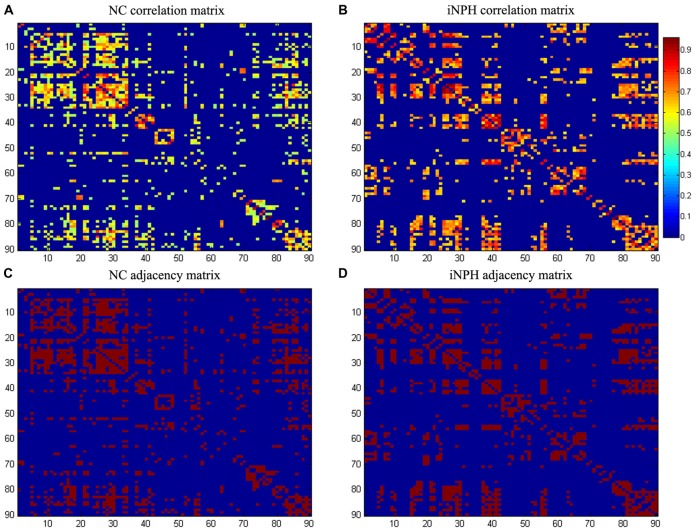
Association matrices and adjacency matrices. Association matrices for the normal-control (NC) group **(A)** and idiopathic normal pressure hydrocephalus (iNPH) group **(B)**. The color-bar shows the strength of the connections. Binary adjacency matrices for the NC group **(C)** and the iNPH group **(D)**, with red indicating the presence of connection and blue the absence of one. These matrices were calculated from maps thresholded at the minimum network density (0.16) in which the networks of both the groups are not fragmented and paths exist between every pair of nodes. Correlations below this threshold and diagonal elements of the matrices are set to zero. The numbers 1–90 on the *x*- and *y-axes* correspond to the gray matter (GM) regions of interest (ROIs) used in the study.

The corrected GM volumes for all 90 ROIs were used to compute a structural correlation network for both the NC and NPH groups, which were 90 × 90 association matrices, R, with each entry *r*_ij_ defined as the Pearson correlation coefficient between the corrected GM volumes of ROI* i* and *j*, across subjects (Figures 1A,B). For each association matrix, a binary adjacency matrix, A, was derived in which *a*_ij_ was considered one if *r*_ij_ was greater than a specific threshold and zero otherwise. The diagonal elements of the constructed association matrix were also set to zero (Figures 1C,D). The resultant adjacency matrix represented a binary undirected graph, G, in which regions i and j were connected if *g*_ij_ was unity. Because of methodological challenges in analyzing and comparing weighted networks (Rubinov and Sporns, 2010, 2011; van Wijk et al., 2010), a graph was constructed with *N* = 90 nodes (anatomical GM ROIs), with a network degree of E equal to number of edges (links) and a network density (cost) of D = E/[N × (N − 1)/2], representing the fraction of present connections to all possible connections. Thresholding the association matrices of different groups at an absolute threshold may yield networks with a different number of nodes and degrees (van Wijk et al., 2010). We thresholded the constructed association matrices at a range of network densities in 0.02 steps (D_min_: 0.10:0.50) and compared the network topologies across that range (Hosseini et al., 2012).

### Network Analyses

#### Global Network Analysis

We calculated the small-world measures of a network originally proposed by Watts and Strogatz (1998), namely the clustering coefficient (C) and characteristic path length (L). The C of a node is a measure of the number of edges between its nearest neighbors. The C of a network is the average of C across nodes and indexes network segregation. The L of a network is the average shortest path length between all pairs of nodes in the network and is the most commonly used measure of network integration (Rubinov and Sporns, 2010). These measures were compared to the corresponding mean values of a null random graph with the same number of nodes, total edges, and degree distribution as the network of interest (Maslov and Sneppen, 2002). Then we obtained the small-world index of the network as [C/C_null_]/[L/L_null_] where, C_null_ and L_null_ are the mean clustering coefficient and the characteristic path length of the null random network (Bassett and Bullmore, 2006). Benchmark random networks generated for functional graphs include topology randomization and correlation matrix randomization (Zalesky et al., 2012).

Modularity is a measure of network segregation that compares the number of connections within modules to the number of connections between modules across the network (Newman, 2006). The whole-brain modularity metric is computed as the sum of the modularity values for each module (Bullmore and Sporns, 2012).

#### Regional Network Analyses

Nodal characteristics, betweenness and degree were quantified and normalized by means of the network, and then compared between the two groups. Nodal betweenness is defined as the fraction of all the shortest paths in the network that pass through a given node and is used to detect important connections. Nodes that bridge disparate parts of the network have a high betweenness (Rubinov and Sporns, 2010). Node degree is defined as the number of connections that a node has with the rest of the network and is considered a measure of the interaction of a node with the network (Hosseini et al., 2013).

Network hubs are crucial components for efficient communication in a network. Hubs not only are considered to be important regulators of information flow, but also play a key role in network resilience to insult (Bullmore and Sporns, 2009). We considered a node as a hub if its betweenness centrality was at least 2 SD higher than mean network centrality (Hosseini et al., 2012).

### Statistics

To test the statistical significance of the between-group differences in network topology and regional network measures, a nonparametric permutation test with 1,000 repetitions was used (He et al., 2008). In each repetition, the calculated residual volumes of each participant were randomly reassigned to one of the two groups so that each randomized group had the same number of subjects as the original groups. Then, an association matrix was obtained for each randomized group. The binary adjacency matrices were estimated by thresholding the association matrices at a range of network densities. The network measures were calculated for all the networks at each density. The differences in network measures between the randomized groups were then calculated, resulting in a permutation distribution of differences under the null hypothesis. The actual between-group difference in network measures was then placed in the corresponding permutation distribution and a two-tailed *P*-value was calculated on the basis of its percentile position (Bernhardt et al., 2011). The nonparametric permutation test inherently accounts for multiple comparisons (*P* < 0.05; Nichols and Hayasaka, 2003). We used area under the curve (AUC; Bernhardt et al., 2011) and functional data analyses (FDA; Hosseini et al., 2012) on global network measures to ensure that differences between the iNPH and NC groups were not driven by differing correlation strengths in regional GM volumes, and further to make the analysis less sensitive to thresholding.

The Graph Analysis Toolbox^2^ (Hosseini et al., 2012) was used to quantify network measures and compare structural networks. Brain Net Viewer^3^ was used for visualizing graphs.

## Results

### Within-Group Global Network Measures

**Figure 2:**
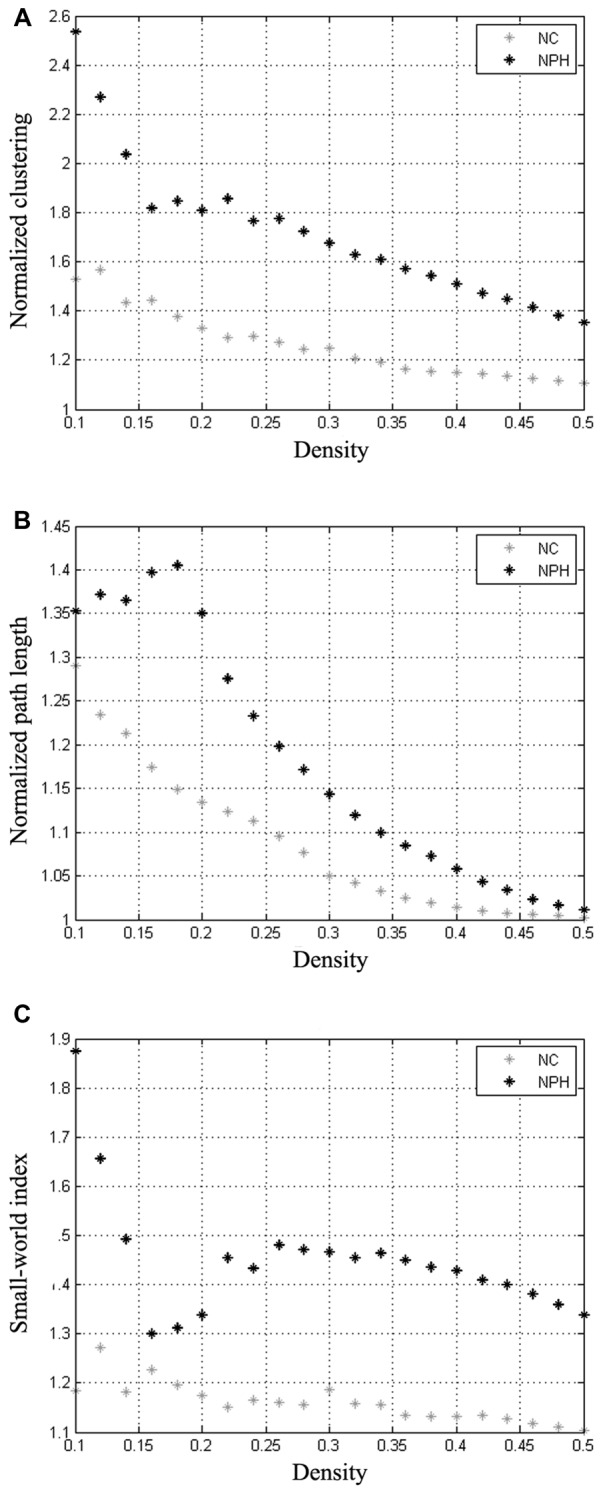
Changes in global network measures as a function of network density. Normalized clustering **(A)**, normalized path length **(B)** and small-world index **(C)** of networks for the iNPH and NC groups. Networks from both the groups follow a small-world organization, with a normalized clustering of greater than 1 and a normalized path length close to 1. The small-world index is bigger than 1 on both networks across all the range of network density.

The minimum network density below which the network was fragmented was 0.16. Global network measures at a range of network densities are shown in Figure 2. The networks of both the groups followed a small-world organization across a wide range of densities. Both the networks had a path length higher than random networks (Figure 2B), while having a clustering coefficient that was much higher than that in random networks (Figure 2A). This pattern results in a small-world index of higher than one across the range of network densities examined (Figure 2C).

### Between-Group Global Network Analysis

**Figure 3:**
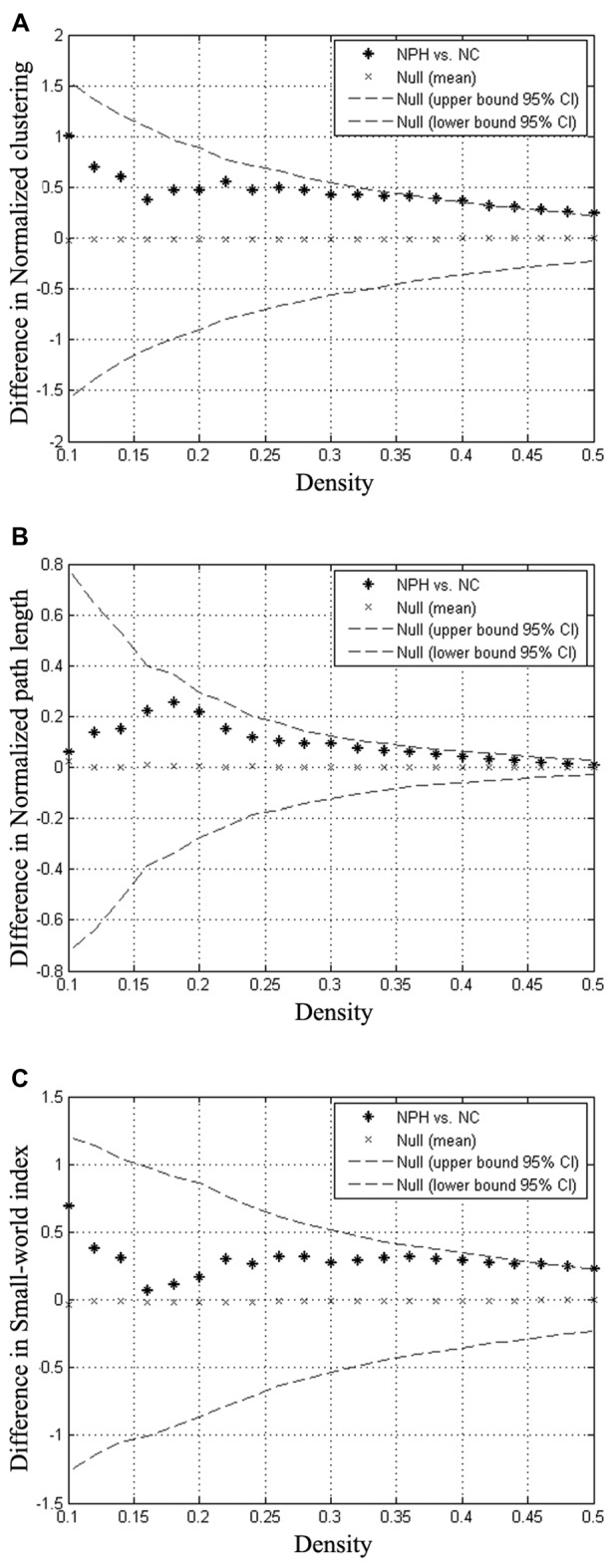
Between-group differences in global network measures as a function of network density. The 95% confidence intervals (dashed lines) and between-group differences (plus markers) in normalized clustering **(A)**, normalized path length **(B)** and small-world index **(C)** for the analysis of iNPH and NC group networks. Differences falling outside the confidence intervals indicate the densities at which the difference between the groups is significant. Positive values indicate densities at which values for iNPH are greater than for NC and negative values indicate the opposite. Relative to the NC network, the normalized clustering, normalized path length and small-world index of the iNPH network were all larger across the entire range of densities.

Compared with the NC group, the iNPH network showed significantly larger normalized clustering at densities ranging from 0.36 to 0.50 (*P* > 0.05; Figure 3A). The iNPH network showed a trend toward larger normalized path length across the whole range of densities, but none of the differences was significant (*P* > 0.05; Figure 3B). This pattern led to a significantly larger small-world index for the iNPH network than the NC network only at densities of 0.48 and 0.5 (Figure 3C). The FDA results showed that the iNPH network had nonsignificant larger normalized clustering (*P* = 0.14), larger normalized path length (*P* = 0.13) and small-world index (*P* = 0.31). The AUC analysis results were consistent with the FDA results; AUC of the iNPH network for the normalized clustering, path length and small-world index were all larger but had no significant difference (*P* = 0.48, 0.12 and 0.96, respectively).

**Figure 4:**
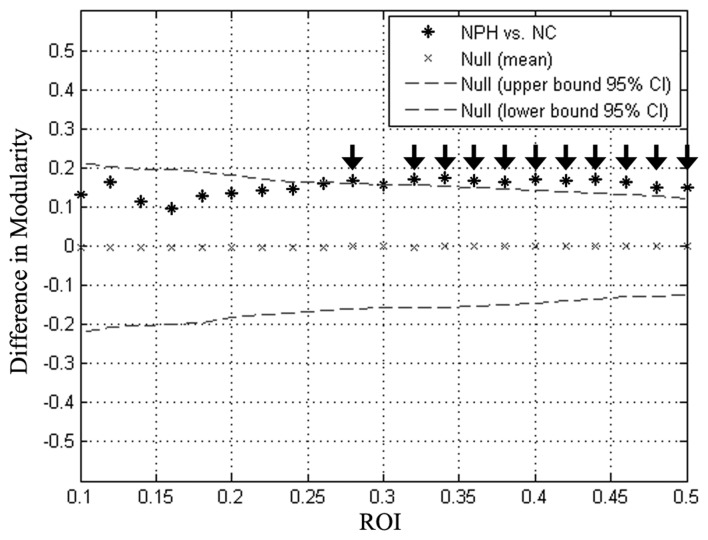
Between-group differences in modularity as a function of network density. Star markers show the difference in modularity between the iNPH and NC networks. Markers falling outside the confidence intervals (dashed lines) with densities ranging from 0.28 to 0.50 (black arrow), excluding 0.30, indicate where the difference in modularity between iNPH and NC network was significant.

Global network modularity was significantly larger in the iNPH network compared with the NC network at all densities ranging from 0.28 to 0.5, except for 0.3 (Figure 4). The FDA and AUC analyses also revealed a significantly larger modularity in the iNPH network (both *P* < 0.05).

#### Regional Network Analyses

**Figure 5:**
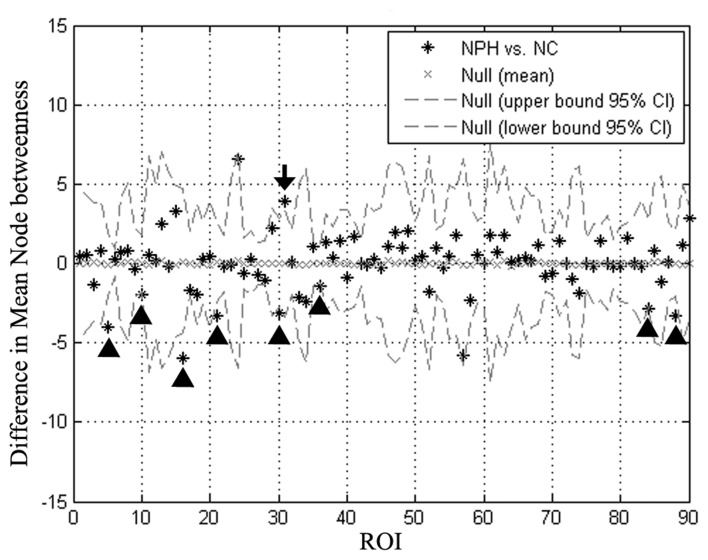
Differences in regional node betweenness for the NC and iNPH networks. Star markers show differences in node betweenness for the iNPH and NC networks. Dashed lines show the 95% confidence intervals. The marker of ROI of number 31 (black arrow) is above the confidence interval, indicating that left anterior cingulum in the iNPH network has a larger betweenness than in the NC network. The markers for ROI of numbers 5, 10, 16, 21, 30, 36, 84 and 88 (black arrowhead) fall below the confidence interval, indicating that these nodes in the iNPH network have a smaller betweenness than in the NC network.

The iNPH network showed significantly smaller betweenness than the NC network in eight nodes including ROI 5 (*P* = 0.01), 10 (*P* = 0.04), 16 (*P* = 0.02), 21 (*P* = 0.02), 30 (*P* = 0.03), 36 (*P* = 0.02), 84 (*P* = 0.04), 88 (*P* = 0.02), which located in the bilateral frontal, right temporal, right insula, and right posterior cingulate regions. Only one region in the left anterior cingulate (ROI 31, *P* = 0.03) showed significantly larger betweenness in the iNPH network (Figure 5).

**Figure 6:**
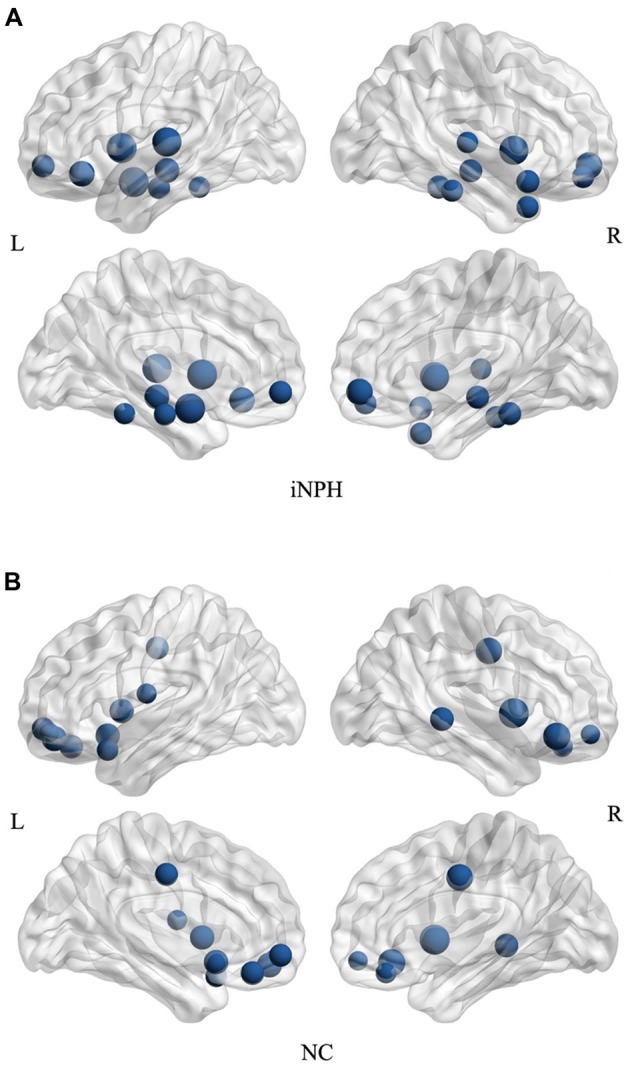
Network hubs for the iNPH and NC groups. All the hubs of the iNPH network **(A)** and the NC network **(B)** were overlaid on the International Consortium for Brain Mapping (ICBM) 152 brain template. The volume of the spheres represents the degree of the corresponding brain region.

The hub analysis revealed 15 hubs in the NC network and 17 hubs in the iNPH network (Figure 6). There were three hubs present in both the networks: the orbital part of the left middle frontal gyrus, and left and right insula. Apart from these hubs, the 14 hubs unique to the iNPH network were primarily located in temporal areas (5 hubs), limbic lobe (3 hubs), frontal areas (3 hubs), fusiform gyrus (2 hubs) and left amygdala. The 12 hubs unique to the NC network were primarily in frontal areas (8 hubs), limbic lobe (2 hubs) and temporal areas (2 hubs).

### Discussion

Earlier work indicated that the altered CSF flow dynamic may be the reason for ventriculomegaly present in the iNPH patients, and speculated that the real root may lie in brain parenchyma (Yin et al., 2017). Most of the previous studies were dedicated to finding one or more anatomical regions responsible for the occurrence of the disease, such as the morphological changes of ventricles, fissures, WM, or GM (Halperin et al., 2015). But none of these abnormal areas, singly or in combination, could explain the typical triad symptoms of iNPH patients, let alone the reason why the symptoms could be released after ventricle shunting. In fact, the distribution of changes detected in the brain of iNPH patients, whether using voxel-based morphometry or the diffusion MR method, was diffuse (Tarnaris et al., 2009). Therefore, taking the brain as whole integrity such as a network and exploring how these changes influence the network may be a more promising direction.

The morphology of the human cerebral cortex has evolved to an optimal neural architecture that supports both modularized and distributed information processing by maximizing the efficiency of information propagation and minimizing wiring costs (Achard and Bullmore, 2007). There are strong correlations in GM morphological features such as volume, density and thickness between various anatomically or functionally linked areas of the human brain (Lerch et al., 2006). A small-world architecture indicates that a network has a combination of both short path length and high clustering (Bullmore and Sporns, 2012). The present study examined the small-world characteristics of GM volume correlation networks of iNPH patients and control subjects. In both the groups, networks had cohesive neighborhoods and short path lengths between regions. Although the iNPH network showed a numerically higher clustering coefficient, short path length and small-world index, the between-group differences were not statistically significant. To the best of our knowledge, this is the first study to investigate the small-worldness of brain networks in iNPH patients. In contrast, Alzheimer’s disease, a more common disease involving cognitive impairment, had received much attention with regard to changes in topological patterns of large-scale cortical networks (He et al., 2009). He et al. (2008) reported a similar, but more significant, difference in small-world characteristics for the patient group compared with controls and proposed that the altered patterns of cortical morphology may relate to the cognitive impairment. The changes in small-world characteristics which we observed in the iNPH patients compared with controls, by contrast, were less obvious.

A more obvious difference was detected in modularity between the iNPH network and the NC network. Modularity is a metric to quantify the extent of a module’s segregation from the rest of the network (Newman and Girvan, 2004). Functionally specialized brain regions with high clustering are termed module (Bullmore and Sporns, 2012). Modular brain network organization is thought to support both the specialized functions through communication within modules and globally integrated functions through communication between network modules. It should be noted that the triad symptoms accompanying iNPH consist of both behavioral and cognitive dysfunctions. There may be at least two abnormal modules or an abnormality in the integration of modules that is responsible for the symptoms exhibited. Furthermore, iNPH is a treatable form of dementia; and modularity was reported as a biomarker that index the potential for adaptive reorganization with intervention (Baniqued et al., 2018). It has been suggested that brain network modularity could be a valuable biomarker that informs the implementation of cognitive interventions. Higher network modularity may represent an optimal brain organization for improving cognitive functioning with training in older adults and traumatic brain injury patients (Arnemann et al., 2015).

We detected fewer hubs in the frontal cortex in the iNPH network than in the NC network. Nodes with high degree typically suggest highly interactive regions that likely participate in numerous functional interactions. The abnormal modularity may be attributed to cortical lesions or subcortical axonal damage (Gratton et al., 2012). Cortical dysfunction in related regions may reflect abnormalities in underlying WM pathways (Catani and Stuss, 2012; Zhou, 2017). Lenfeldt et al. (2011) reported lesions in anterior frontal WM using diffusion tensor imaging (DTI), and suggested that these were related to the motor symptoms of iNPH. Moreover, on the basis of voxel-wise analysis of DTI, various patterns of WM changes were detected in regions bordering GM, such as corpus callosum, periventricular WM, the internal capsule and so on (Hattori et al., 2012). This may explain why several nodes of the iNPH network showed significantly decreased node betweenness, whereas only one node increased its betweenness, as well as the changes of modularity. Thus, we hypothesized that modularity changes may be the cause of iNPH, and modularity analysis was a potential biomarker to select shunting responsive patients.

There were two limitations to this study. First, it was not possible to correlate the network measures of each subject with their individual symptom severity score using this method. In the future, a longitudinal evaluation of a group of patients could be conducted to examine such correlations. Second, due to the low incidence of iNPH, the number of patients was not sufficient to divide into shunting-responsive and nonresponsive groups. However, this preliminary study detected abnormalities in structural networks in iNPH. Brain network analysis was a promising method to study neurological disorder diseases. Meanwhile, this reversible dementia was the ideal disease model to study cognition of humans. A network connectivity study using DTI or BOLD-MRI could be a promising approach to clarify the pathological mechanisms underlying iNPH. Furthermore, it may be fruitful to employ network modularity analysis to predict the outcome of shunt treatment in iNPH patients.

## Author Contributions

LY, JZ, HY and YY: guarantors of integrity of entire study and manuscript editing. All authors: study concepts/study design or data acquisition or data analysis/interpretation, approval of final version of submitted manuscript and agrees to ensure any questions related to the work are appropriately resolved. LY, YY and JZ: manuscript drafting or manuscript revision for important intellectual content. LY, XH, JT and JZ: literature research. JZ, LY and JT: clinical studies. LY, YZ and CL: statistical analysis.

## Conflict of Interest Statement

The authors declare that the research was conducted in the absence of any commercial or financial relationships that could be construed as a potential conflict of interest. The reviewer CA and handling Editor declared their shared affiliation at the time of the review.

## Footnotes

^1^https://web.mit.edu/swg/software.htm

^2^https://redcap.stanford.edu/surveys/?s=WTSR8K

^3^https://www.nitrc.org/projects/bnv/
